# SARS‐CoV‐2 Viral Load and Cytokine Dynamics Profile as Early Signatures of Long COVID Condition in Hospitalized Individuals

**DOI:** 10.1111/irv.70068

**Published:** 2025-01-12

**Authors:** Jacobo Alonso Domínguez, Inés Martínez Barros, Irene Viéitez, Mercedes Peleteiro, Beatriz Calderón‐Cruz, José A. González‐Nóvoa, Alexandre Pérez González, Virginia Leiro Fernández, Aida López López, Eva Poveda López

**Affiliations:** ^1^ Virology and Pathogenesis Galicia Sur Health Research Institute (IIS Galicia Sur), SERGAS‐UVIGO Vigo Spain; ^2^ Genomics Unit Galicia Sur Health Research Institute (IIS Galicia Sur), SERGAS‐UVIGO Vigo Spain; ^3^ CINBIO Universidade de Vigo Vigo Spain; ^4^ Statistics and Methodology Unit Galicia Sur Health Research Institute (IIS Galicia Sur), SERGAS‐UVIGO Vigo Spain; ^5^ AI Platform Galicia Sur Health Research Institute (IIS Galicia Sur), SERGAS‐UVIGO Vigo Spain; ^6^ Departamento de Tecnología Electrónica Universidade de Vigo Vigo Spain; ^7^ Internal Medicine Department Complexo Hospitalario Universitario de Vigo (CHUVI), Sergas Vigo Spain; ^8^ Pneumology Department Complexo Hospitalario Universitario de Vigo (CHUVI), Sergas Vigo Spain; ^9^ NeumoVigo I+i Research Group Galicia Sur Health Research Institute (IIS Galicia sur), SERGAS‐UVIGO; CIBERES, ISCIII Vigo Spain

**Keywords:** cytokines, long COVID condition, SARS‐CoV‐2, viral load

## Abstract

**Background:**

The global pandemic caused by SARS‐CoV‐2 has resulted in millions of people experiencing long COVID condition, a range of persistent symptoms following the acute phase, with an estimated prevalence of 27%–64%.

**Materials and Methods:**

To understand its pathophysiology, we conducted a longitudinal study on viral load and cytokine dynamics in individuals with confirmed SARS‐CoV‐2 infection. We used reverse transcriptase droplet digital PCR to quantify viral RNA from nasopharyngeal swabs and employed multiplex technology to measure plasma cytokine levels in a cohort of people with SARS‐CoV‐2 infection. Our study included individuals with long COVID condition and those without, all of whom had at least three nasopharyngeal and plasma samples collected within 55 days after diagnosis of SARS‐CoV‐2 infection.

**Results:**

Individuals affected with long COVID symptoms had delayed viral clearance and lower viral loads at diagnosis compared to those without symptoms. Additionally, cytokine analysis revealed variations in IL‐18, MIG, and IP‐10 levels, with delayed normalization in individuals affected by long COVID syndrome. Correlation analysis indicated associations between viral load and IP‐10 and interrelations among cytokines IL‐1β, IL‐18, MIG, and IP‐10.

**Conclusion:**

Our study provides insights into the association between nasopharyngeal viral load, cytokine dynamics, and the development of long COVID syndrome, providing an early signature of this condition.

## 1 Introduction

Four years after a pandemic that affected the entire world, the consequences of the SARS‐CoV‐2 are still seen in the form of a heterogeneous set of symptoms that indiscriminately affect the different systems of the human body: the long COVID condition. The estimated prevalence of this condition ranges from 27% to 64%, depending of the definition used [1], which means that, taking as a reference the World Health Organization (WHO) figures of 775 million people infected worldwide, more than 300 million people would suffer from this condition.

The terms “long COVID” or “long COVID syndrome” are employed broadly to characterize the persistence or recurrence of symptoms over an extended period. At first, many studies developed their own definition of this condition, until the WHO proposed its definition of “the continuation or development of new symptoms 3 months after the initial SARS‐CoV‐2 infection, with these symptoms lasting for at least 2 months with no other explanation” [2]. However, this definition provides a large umbrella under which many different symptomatologies can fit.

There are countless studies that enumerate the different possible manifestations of this condition, but all of them converge in the lack of knowledge of the pathophysiology, for which we provide new data. These manifestations are highly variable and sometimes the symptoms are nonspecific, ranging from neurological (headache, sleep problems, depression, and ageusia) to thoracic (chest pain and cough), musculoskeletal (myalgias and arthralgias), or general (asthenia and fever) [3].

Despite all the research done in biomarkers associated with this condition [4], the absence of a more precise or accurate molecular definition of this symptomatology is not the only knowledge gap. Understanding the pathophysiology of a disease is always a benefit to elucidate the best forms of prevention or treatment to avoid or minimize an outcome that may condition the person's life in the future. Previous studies have identified several risk factors for the development of long COVID syndrome symptomatology, such as being female, high blood pressure, obesity, a prior diagnosis of psychiatric disease, or immunosuppression. On the other hand, aging does not increase the risk for long COVID syndrome [5, 6].

Thus, we longitudinally evaluate both nasopharyngeal viral load (VL) and proinnflamatory cytokines involved in the immune response to SARS‐CoV‐2 infection. We also evaluated the correlations between these biomarkers among people suffering from long COVID condition and controls.

## 2 Material and Methods

### 2.1 Study Population

Nasopharyngeal exudates and plasma samples were selected from individuals who belonged to the COVID cohort of the Instituto de Investigación Sanitaria Galicia Sur (https://www.iisgaliciasur.es/apoyo‐a‐la‐investigacion/cohorte‐covid19/), which is composed of persons diagnosed with SARS‐CoV‐2 by PCR (Cobas® SARS‐CoV‐2 test‐*Roche Diagnostics*, NJ, USA, or Allplex SARS‐CoV‐2 Assay‐*Seegene Inc*). For the current study, we chose both individuals who had long COVID symptomatology (following the definition established by the WHO) and those whose symptomatology had resolved within 1 month after infection. In addition, all individuals had to have at least three follow‐up points (one of them being baseline, i.e., closest to the day of confirmation of infection) of both paired nasopharyngeal exudate and plasma samples obtained within the first 55 days after diagnosis of SARS‐CoV‐2.

### 2.2 RNA Extraction of SARS‐CoV‐2

Viral RNA extraction was carried out from 140 μL of nasopharyngeal swab samples utilizing the QIAmp Viral RNA Mini Kit (QIAGEN, Hilden, Germany) and the automated QIAcube System (QIAGEN, Hilden, Germany). The elution step was performed with 50 μL of buffer according to the manufacturer's protocol. Positive (EDX SARS‐CoV‐2 Standard, Exact Diagnostics, Fort Worth, TX, USA) and negative (EDX SARS‐CoV‐2 Negative, Exact Diagnostics, Fort Worth, TX, USA) controls underwent the same extraction procedure.

### 2.3 Droplet Digital PCR Analysis

SARS‐CoV‐2 VL was assessed using reverse transcriptase droplet digital PCR (RT‐ddPCR), employing the one‐step reverse transcription method (One‐Step RT‐ddPCR Advanced Kit for Probes, Bio‐Rad Laboratories, Hercules, CA, USA) and a triplex probe assay for PCR amplification (2019‐nCoV CDC ddPCR Triplex Probe Assay, Bio‐Rad Laboratories, Hercules, CA, USA). This assay targeted two regions of the SARS‐CoV‐2 nucleocapsid gene (N1 and N2) as well as the human Rnase P gene (RPP30). Each reaction mixture comprised 5.5 μL of SARS‐CoV‐2 RNA sample, following the manufacturer's instructions, and all samples were tested in duplicate. Data analysis utilized the QuantaSoft Analysis Pro Software (v. 1.0.596, Bio‐Rad Laboratories, Hercules, CA, USA), presenting results as copies per microliter of 1x ddPCR reaction. To facilitate comparison across samples, all VL values were recalibrated to copies per milliliter of swab, with a sensitivity threshold set at 100 copies per milliliter. Accuracy of absolute viral RNA quantification was evaluated in a previous study of the group through linear regression analysis of twofold serial dilutions of the positive control [7].

### 2.4 Cytokine Quantification

Plasma concentrations of cytokines were determined using Luminex xMAP (Multi‐Analyte Profiling) technology. Interleukin (IL)‐1β, IL‐18, interferon gamma‐induced monokine (MIG), and interferon gamma‐induced protein 10 (IP‐10) were analyzed using a microsphere‐based multiplex immunoassay (MILLIPLEX® Human Cytokine/Chemokine/Growth Factor Panel A, Merck KGaA, Darmstadt, Germany) and read on a Luminex MAGPIX analyzer, located at the CINBIO (Centro de Investigación en Nanomateriais e Biomedicina) facilities, according to manufacturer's instructions.

### 2.5 Statistical Analysis

Descriptive analyses were presented as frequencies and percentages for categorical variables and as medians and interquartile ranges (IQR) for continuous variables. Due to the temporal dispersion of sample collection relative to the date of the positive PCR, the value of each measurement in each time became a case within a continuous time frame, resulting in 104 measurements of both VL and each cytokine. Kaplan–Meier survival analysis was used to assess the time to viral clearance in nasopharyngeal samples or, in the case of cytokines, return to nonpathological plasmatic values. From these curves, we also compared the dynamics of the different analytes between individuals who subsequently presented long COVID symptoms and those who did not by the method of log‐rank test (Mantel‐Cox). The Shapiro–Wilk test was used to test the normality of the distributions in each of the groups, where it was observed that both VL and cytokines had nonparametric distributions in one or both groups except IL‐18. However, due to the small sample (12 and 18 patients in each group), a nonnormal distribution of the data is assumed, so the Mann–Whitney test was conducted to check for differences in baseline VL (near PCR diagnosis) between those who would develop long COVID condition and those who would not. Spearman's correlation was employed to examine correlations between different variables.

Statistical analyses were conducted using SPSS (v26, IBM Corp, Armonk, NY, USA) and GraphPad Prism (v8.0.1, GraphPad Software, San Diego, CA, USA), with a significance level set at *p* < 0.05.

### 2.6 Smoothing Splines

For addressing the interpolation challenge in our study, our objective was to create a curve that accurately captures the dynamics of VL from SARS‐CoV‐2 diagnosis up to 1 month postinfection. However, traditional interpolation methods might not be suitable, particularly in the presence of noisy data. Hence, we aimed to construct a smooth curve, denoted as *g*(*x*), which could effectively approximate the input data without necessarily passing through every individual point.

To achieve this, we utilized the *scipy.interpolate* module, which facilitates the construction of smoothing splines. These splines are based on the FITPACK Fortran library developed by P. Dierckx.

More specifically, given the data arrays *x* and *y*, along with an array of nonnegative weights represented by *w*, we sought a spline function *g*(*x*) that met the condition $\sum_{j}{\left[w_{j}\left(g\left(x_{j}\right)-y_{j}\right)\right]}^{2}\leq s$. These conditions were determined by an input parameter denoted as *s*, which controlled the balance between the smoothness of the resulting curve *g*(*x*) and the accuracy of the data approximation, as measured by the differences between *g* (*x*_*j*_) and *y*_*j*_.

## 3 Results

A total of 30 individuals met the established criteria, all of them having between three and five measurement points within the first 55 days after the diagnosis, in addition to the baseline sample. This resulted in a total of 104 determinations for each of the molecules analyzed. Of all these determinations, 41 were from individuals with long COVID symptomatology (*n* = 12), whereas the remaining 63 determinations were from persons who had no symptoms (*n* = 18) beyond those experienced during infection. Table 1 shows the clinical/epidemiological data of this study population, where none of the comorbidities show significant differences between both groups. All individuals in the study required hospital admission; however, there were differences in the median number of days of hospitalization, where the long COVID group had a hospital stay of 7 [6.25–12] days and the nonlong COVID group was hospitalized for 10.5 [8.25–23.25] days (*p* = 0.037). Of all of them, 36.4% were considered critical because they had a ratio between arterial oxygen pressure and inspired oxygen fraction (PaFi) < 200 mmHg on admission.

**TABLE 1:** Clinical and epidemiological characteristics of the study population.

	Nonpost COVID	Post COVID	*p*
Patients in follow‐up	18	12	
Demographics			
Age, median [IQR]	61 [52.3–75]	58.5 [48.3–70]	0.378
< 55, *n* (%)	6 (33.3)	4 (33.3)	0.650
≥ 55, *n* (%)	12 (66.7)	8 (66.7)	
Male sex	14 (77.8)	9 (75)	0.597
Comorbidities, *n* (%)			
Obesity	8 (44.4)	5 (41.7)	1.000
Hypertension	7 (38.9)	3 (25)	0.694
Chronic obstructive pulmonary disease	2 (11.1)	3 (25)	0.364
Diabetes mellitus	2 (11.1)	2 (16.7)	0.622
Asthma	2 (11.1)	0 (0)	0.503
HIV infection	0 (0)	1 (8.3)	0.400
Chronic kidney disease	1 (5.6)	0 (0)	1.000
Chronic inflammatory disease	1 (5.6)	1 (8.3)	1.000
Severity			
Moderate	14 (77.8)	8 (66.7)	0.396
Critical	4 (22.2)	4 (33.3)	
Days from symptoms onset to SARS‐CoV‐2 confirmation	5.5 [1.5–11.25]	3 [1–10]	0.478
Days of hospitalization	10.5 [8.25–23.25]	7 [6.25–12]	**0.037**
Admission to ICU	7 (38.9)	2 (16.7)	0.261
Invasive mechanical ventilation	5 (27.8)	1 (8.3)	0.204
Tobacco use			
Smoker	3 (16.7)	1 (8.3)	0.196

*Note:* Bold font indicates *p* value < 0.05.

### 3.1 Correlations

When correlating the different markers assessed, SARS‐CoV‐2 VL correlates with IP‐10 (correlation coefficient [CC]: 0.486, *p* < 0.001). Between the cytokines, IL‐1β had direct correlations with IL‐18 (CC 0.309, *p* = 0.001), IP‐10 (CC 0.240, *p* = 0.014), and MIG (CC 0.355, *p* < 0.001). Regarding IL‐18, Spearman's test showed correlations with IP‐10 (CC 0.421, *p* < 0.001) and MIG (CC 0.440, *p* < 0.001). Finally, IP‐10 also correlates with MIG (CC 0.602, *p* < 0.001). In addition, lymphocyte levels correlated inversely with both IP‐10 (CC −0.445, *p* = 0.014) and MIG (CC −0.492, *p* = 0.006) (Table S1 and Figure S1a–g).

When comparing the VL at the diagnosis with the number of symptoms 1 month after the onset of the infection, the correlation coefficient showed an inverse correlation of −0.327 (*p* = 0.077).

### 3.2 SARS‐CoV‐2 Viral Load and Cytokine Dynamics Shows Differences Between Those With and Without Long COVID Symptomatology

**FIGURE 1:**
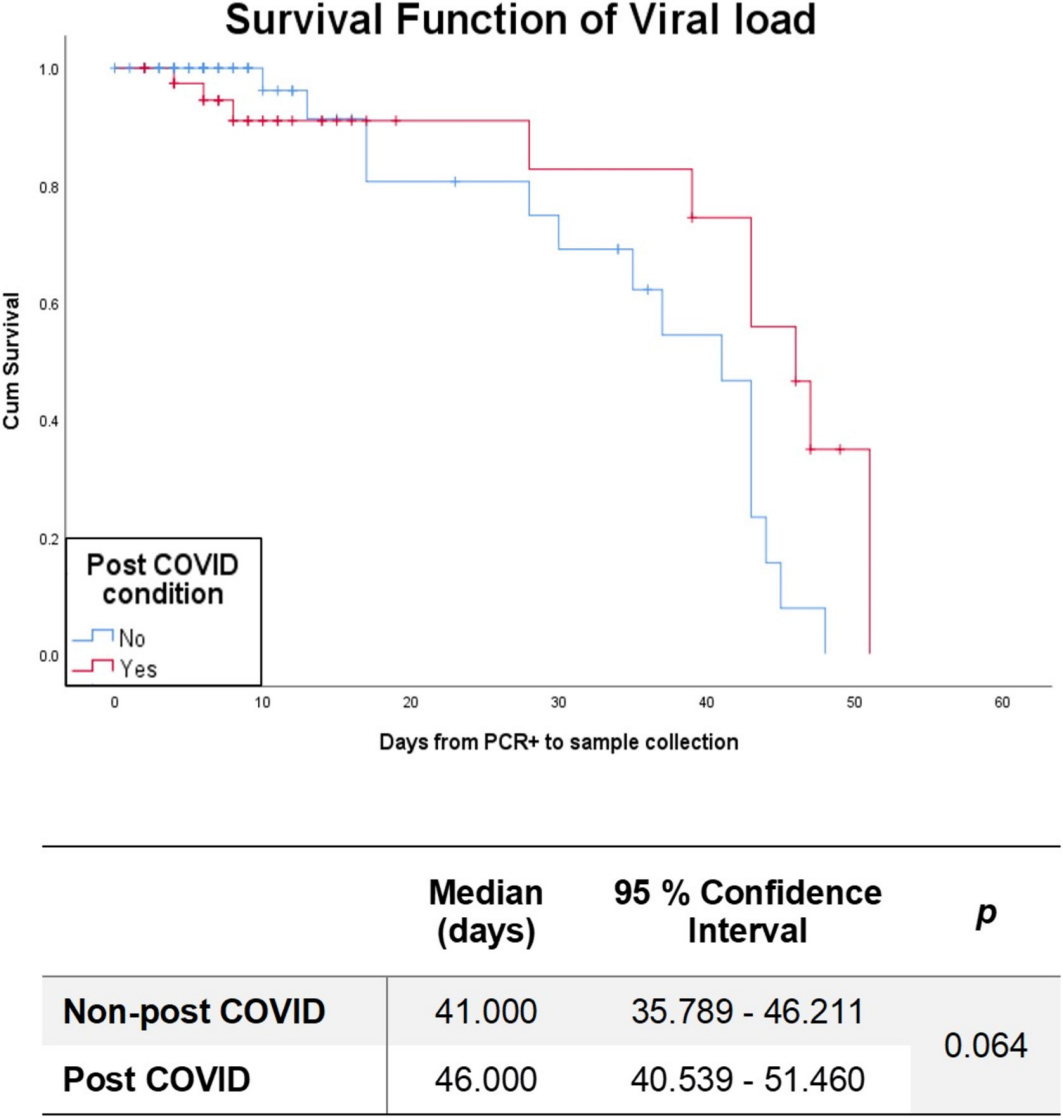
Survival function of viral load in nonlong COVID and long COVID and their respective statistical values.

A survival curve was obtained to establish the median time it takes for an individual to obtain a negative PCR for SARS‐CoV‐2, using all 104 sample points for VL, which was 43 days from positive PCR (Figure 1).

When these data were disaggregated among individuals who had long COVID symptomatology 3 months after infection, those who developed this condition took a median of 46 days to become negative, whereas those who did not took a median of 41 days (*p* = 0.064) (Figure S2a).

When comparing the VL of the long COVID group with the nonlong COVID group at diagnosis, the former have a median baseline VL of 6985 [395–587,142] copies/ml, whereas the latter have a median baseline VL of 148,285 [14,171–9,768,214] copies/ml (*p* = 0.059).

This product limit estimator was also used for the each of the cytokines analyzed, to see how much time it would need for each one to reach its normal values. To establish these normal values, data obtained in a previous study using a control cohort of individuals without SARS‐CoV‐2 infection were used [8].

In its survival function, IL‐1β showed no significant differences between the two groups for the number of days at which half of the determinations returned to normal values (12 days for long COVID group and 12 days for nonlong COVID group; *p* = 0.232). Both IL‐18 and MIG showed differences in their values between long COVID and nonlong COVID groups. In the case of IL‐18, it took 22 days for half of the determinations to return to normal values in the long COVID group, whereas in the nonlong COVID group, this time was 17 days (*p* = 0.049) (Figure S2b). Similarly for MIG, the median days to recovery the normal values were 15 days in long COVID and 10 days in nonlong COVID (*p* = 0.037) (Figure S2c). In case of IP‐10, no differences (*p* = 0.082) were observed between the two groups in normalizing its values after the infection (Figure S2d).

### 3.3 Smoothing Splines Illustrate the Common Dynamics in Viral Load and Proinflammatory Cytokines

**FIGURE 2:**
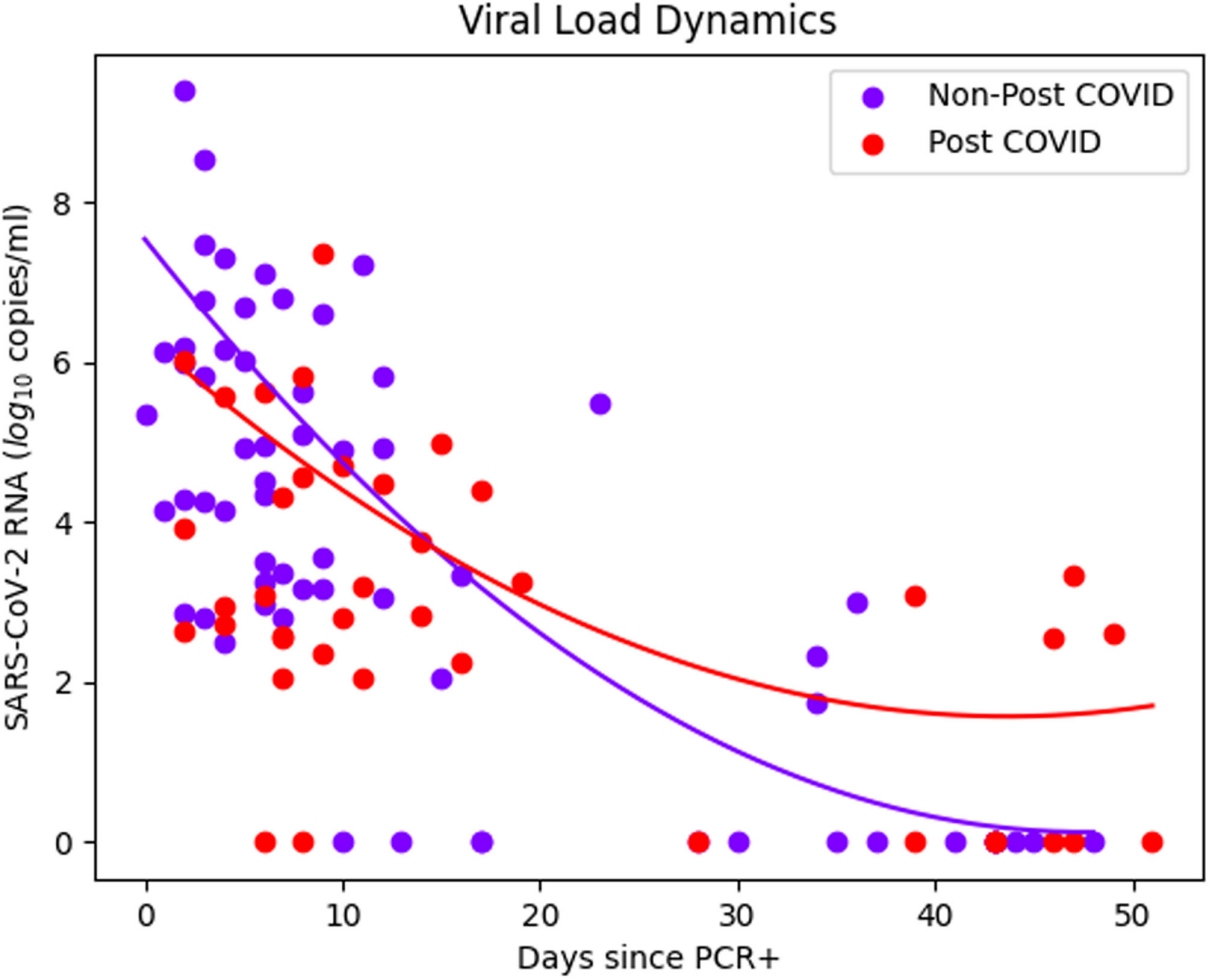
Viral load values detected by ddRT‐PCR in nasopharyngeal samples from SARS‐CoV‐2 infected individuals stratified by the presence or absence of long COVID condition. The line represents the trend of viral load using smoothing splines.

The dynamics of VL and cytokines have been represented using smoothing splines to obtain a second‐order curve that effectively represented the imputed data. Figure 2 shows the comparison of SARS‐COV‐2 RNA VL dynamics between long COVID and nonlong COVID groups, where people that would end up developing long COVID symptomatology 3 months after the onset of the infection had lower levels of SARS‐CoV‐2 RNA at diagnosis, and it takes longer to become negative for PCR in nasopharyngeal exudate.

Regarding cytokine dynamics, both IL‐1β and IL‐18 (Figure S3a,b) show a similar trend to that of VL, in which values at diagnosis appeared to be lower in the long COVID group, although they remained at practically the same levels, which is not the case in nonlong COVID, whose values do appear to decrease over time. IP‐10 (Figure S3c) showed the sharpest decrease of all cytokines, with both groups starting from similar levels; however, individuals who will develop the long COVID condition tend to have higher levels towards the end of the study. Finally, MIG (Figure S3d) also showed a decrease in its levels in both groups as time elapsed from the day of diagnosis; nevertheless, the levels of this cytokine in the long COVID group remained above those of the nonlong COVID group throughout the study. However, due to the idiosyncrasy of smooth splines, we were not able to perform a statistical test to compare both curves.

## 4 Discussion

In this study, we examined the differences between people who, after being infected with SARS‐CoV‐2, developed long COVID symptomatology and those who resolved the infection without symptoms beyond those experienced during the infection. The study population consisted of individuals who met criteria to be categorized as having long COVID symptomatology and individuals who did not fit this definition, all with a nasopharyngeal exudate and plasma sample at the time of diagnosis of SARS‐CoV‐2 infection and between two and four more follow‐up samples (both exudate and plasma) during the 55 days following confirmation of SARS‐CoV‐2 by PCR+. Of the resulting 30 individuals, 12 developed long COVID symptomatology, whereas 18 had no symptoms beyond those developed during infection, resulting in a total of 104 determinations of both VL and plasma cytokines.

Regarding their clinical and epidemiological characteristics, no differences were observed between the two groups except when comparing the number of days of hospitalization, where the nonlong COVID population showed a higher median number of days of hospitalization than the long COVID, which could indicate that the days of hospitalization and, therefore, the initial severity of the infection would not necessarily imply the subsequent development of the long COVID condition.

When it comes to whether VL plays a role in the outcome of SARS‐CoV‐2 infection, there is still no consensus, with some studies claiming that the VL at diagnosis is higher in those who had worse prognosis [9], whereas others claim that there is no correlation between viral load and infection‐associated outcome [10]. There are even studies that establish an inverse correlation between the VL and the severity of COVID‐19 and even an inverse correlation between this VL and the duration of symptoms [11]. Our study suggests that the initial viral load at diagnosis is not associated to the development of long COVID or to the time required for the viral load to become undetectable. However, it appears to be relevant in relation to symptomatology, where we observed an inverse correlation with the number of symptoms.

In the case of cytokines, their relationship with infection is more unequivocally established, and IL‐1β, IL‐18, MIG, and IP‐10 have been related to COVID‐19 [8, 12]. All of them have high values at diagnosis that decrease as the infection resolves, both in the group that maintains symptomatology (or develops a new one) and in those who do not suffer from this condition. In fact, we previously postulated IL‐1β as a differential cytokine between both groups 1 month after infection [8]. However, in these new extended data obtained in this study, it is observed that despite maintaining a uniform decrease, when comparing the long COVID group with the nonlong COVID group, both IL‐18 and MIG have differences in the time required to return to nonpathological cytokine values in each of the groups. Something similar occurs with IP‐10, because the time difference for normalization of its values between both groups approaches statistical significance. As a regulator of antiviral immunity through its leukocyte chemoattractant activity, IP‐10 has shown association with VL in diverse viral infections, from respiratory virus [13] to HIV [14]. In accordance with the existing published data [15], we also observed in our study a correlation between VL and IP‐10 levels. Similarly, we previously described this correlation between VL and IP‐10 in the context of the HIV infection; in this case, this correlation was also observed for MIG. Interestingly, both IP‐10 and MIG are induced by interferon‐gamma (IFN‐γ), showing a similar chemotactic role of immune cells and a correlation with IL‐18, which is consistent with previous studies because IL‐18 can induce the release of IFN‐γ and, consequently, the induction of IP‐10 and MIG [15].

IL‐1β showed correlations with all other cytokines. This is reasonable when considering that IL‐1β shares synthesis pathway with IL‐18 through the NLRP3 inflammasome, which has been shown to undergo considerable activation in SARS‐CoV‐2 infection [16]. In addition, it has been seen how IL‐1β can favor the release of IP‐10 [17, 18].

When using the smoothing splines to illustrate the tendencies of the VL and cytokines, all of them share a similar pattern. The curves corresponding to the long COVID group start from lower values at baseline of VL and cytokines, and as time elapses after the onset of infection, they progressively descend as the nonlong COVID curves do. However, during follow‐up, all long COVID dynamics (except for MIG) reverse their figures with respect to nonlong COVID, concluding the follow‐up period with higher values.

This study has certain limitations that should be acknowledged. First, all participants were hospitalized; therefore, the results obtained cannot be extrapolated to individuals with SARS‐CoV‐2 infection who have not been hospitalized. Second, the sample size is relatively small, which could impact the robustness of the conclusions. Nevertheless, the study successfully identified early signatures of long COVID, which would be valuable to validate in other cohorts.

In conclusion, our study provides an early signature that highlights the different profiles in VL and cytokine dynamics from the onset of infection and the following weeks between individuals who develop long COVID and those who do not. Correlations between cytokines such as IP‐10, MIG, and IL‐18 indicate intricate immune responses to SARS‐CoV‐2 infection, potentially influencing disease outcomes. These findings underscore the need for further research to elucidate the mechanisms underlying long COVID condition and identify therapeutic targets to mitigate its impact.

## Author Contributions

**Jacobo Alonso Domínguez:** formal analysis, investigation, methodology, writing – original draft. **Inés Martínez Barros:** investigation. **Irene Viéitez:** investigation. **Mercedes Peleteiro:** investigation. **Beatriz Calderón‐Cruz:** methodology. **José A. González‐Nóvoa:** methodology. **Eva Poveda López:** conceptualization, investigation, writing – original draft.

## Conflicts of Interest

The authors declare no conflicts of interest.

### Peer Review

The peer review history for this article is available at https://www.webofscience.com/api/gateway/wos/peer‐review/10.1111/irv.70068.

## Supporting information


**Figure S1.** Graphs corresponding to Spearman’correlations of (A) SARS‐CoV‐2 VL and IP‐10, (B) IL‐1β and IL‐18, (C) IL‐1β and IP‐10, (D) IL‐1β and MIG, (E) IL‐18 and IP‐10, (F) IL‐18 and MIG and (G) IP‐10 and MIG.

**Figure S2.** Survival function in non‐long COVID and long COVID individuals and their respective statistical values of (A) IL‐1β, (B) IL‐18, (C) MIG and (D) IP‐10.

**Figure S3.** Plasma values from SARS‐CoV‐2 infected individuals stratified by the presence or absence of long COVID condition of (A) IL‐1β, (B) IL‐18, (C) IP‐10 and (D) MIG. The lines represent the trend of each cytokine using smoothing splines.

**Table S1.** Values of correlations between viral load and different cytokines.

## Data Availability

The data that support the findings of this study are available in the Supporting Information of this article.
