# Supplementary figures and images for: SARS‐CoV‐2 Viral Load and Cytokine Dynamics Profile as Early Signatures of Long COVID Condition in Hospitalized Individuals
Source: Influenza Other Respir Viruses. 2025 Jan 12;19(1):e70068. doi: 10.1111/irv.70068 (PMC11725401; doi:10.1111/irv.70068)

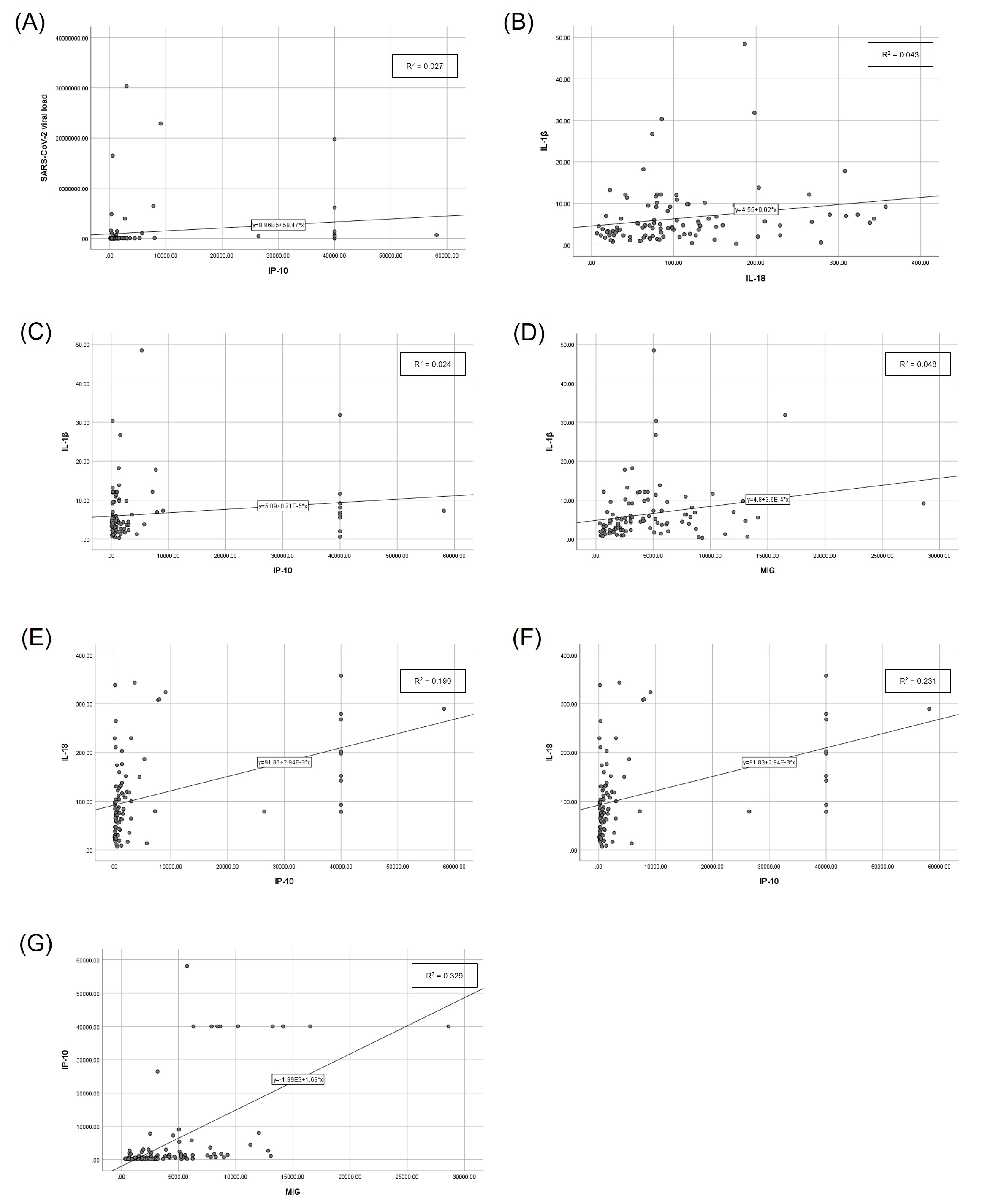

Supplement: Supplementary file 1 — Figure S1. Graphs corresponding to Spearman’correlations of (A) SARS‐CoV‐2 VL and IP‐10, (B) IL‐1β and IL‐18, (C) IL‐1β and IP‐10, (D) IL‐1β and MIG, (E) IL‐18 and IP‐10, (F) IL‐18 and MIG and (G) IP‐10 and MIG. [file IRV-19-e70068-s002.tif]

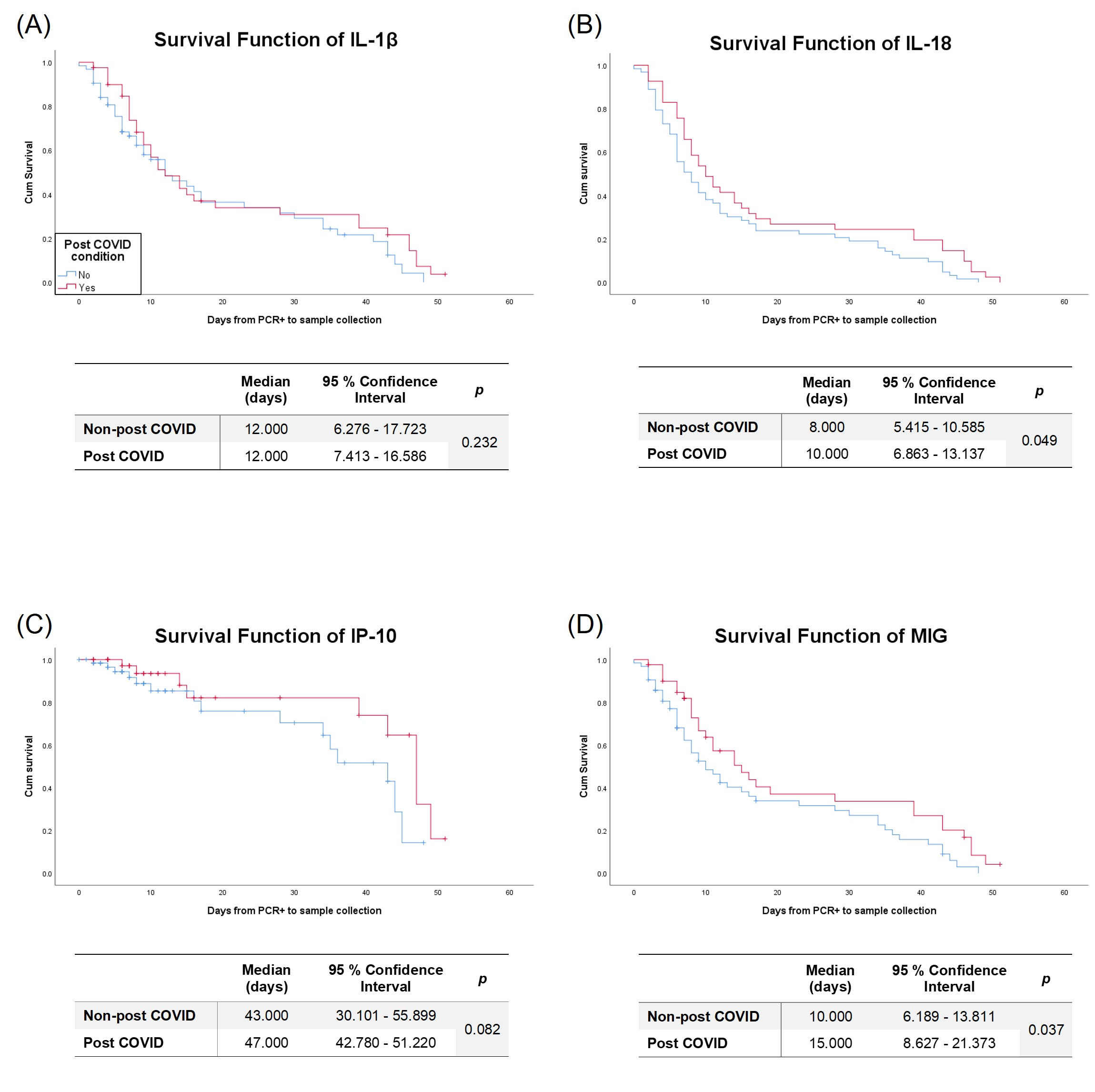

Supplement: Supplementary file 2 — Figure S2. Survival function in non‐long COVID and long COVID individuals and their respective statistical values of (A) IL‐1β, (B) IL‐18, (C) MIG and (D) IP‐10. [file IRV-19-e70068-s003.tif]

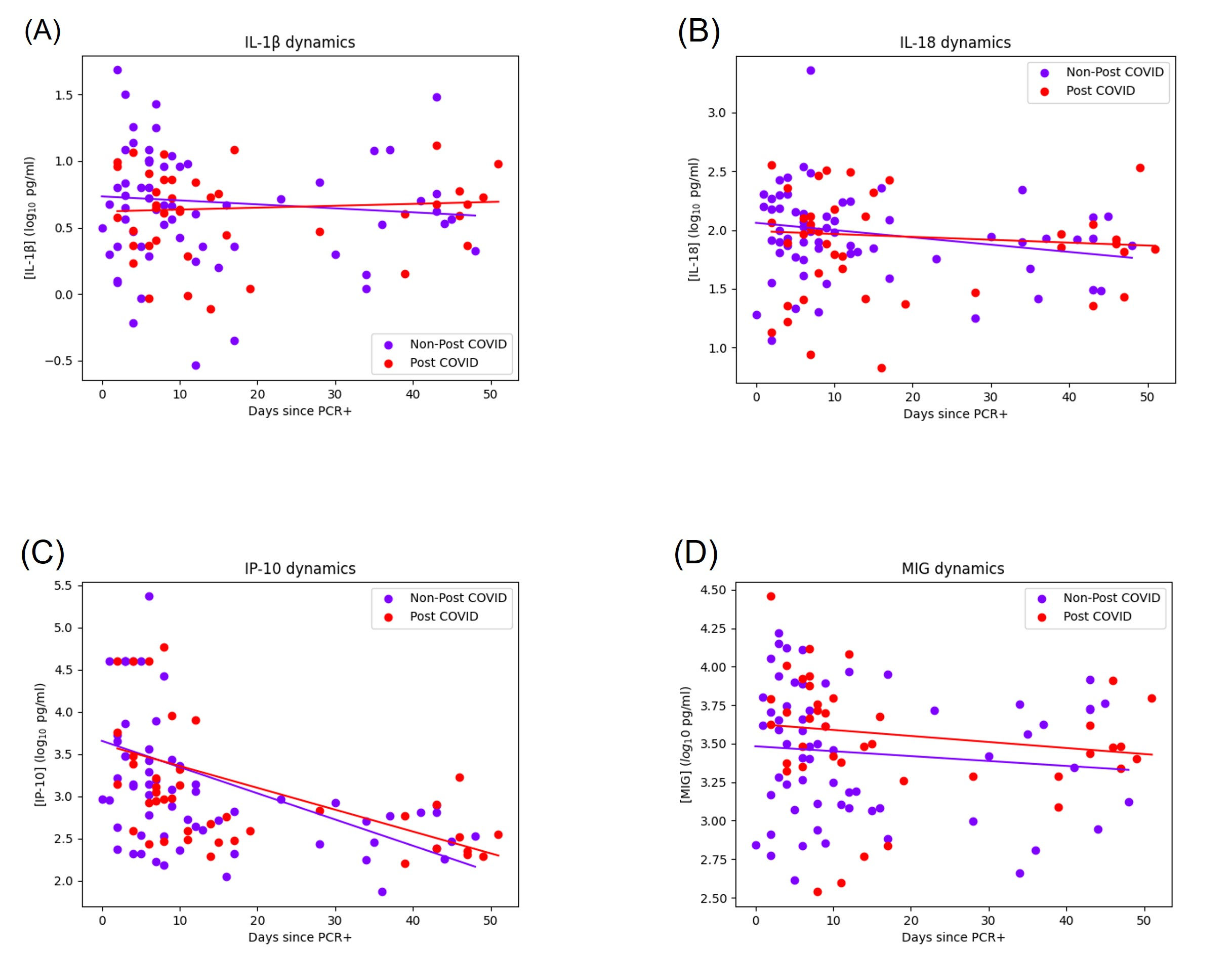

Supplement: Supplementary file 3 — Figure S3. Plasma values from SARS‐CoV‐2 infected individuals stratified by the presence or absence of long COVID condition of (A) IL‐1β, (B) IL‐18, (C) IP‐10 and (D) MIG. The lines represent the trend of each cytokine using smoothing splines. [file IRV-19-e70068-s001.tif]
