# Supplementary material for: SARS‐CoV‐2 Viral Load and Cytokine Dynamics Profile as Early Signatures of Long COVID Condition in Hospitalized Individuals
Source: Influenza Other Respir Viruses. 2025 Jan 12;19(1):e70068. doi: 10.1111/irv.70068 (PMC11725401; doi:10.1111/irv.70068)
Supplement: Supplementary file 4 — Table S1. Values of correlations between viral load and different cytokines. [file IRV-19-e70068-s004.docx]

|  | SARS-CoV-2 viral load | IL-1β | IL-18 | IP-10 | MIG |
| --- | --- | --- | --- | --- | --- |
| SARS-CoV-2 viral load | 1.000 | 0.124  *p* = 0.214 | 0.192  *p* = 0.053 | **0.454**  *p* < 0.001 | 0.038  *p* = 0.706 |
| IL-1β |  | 1.000 | **0.309**  *p* = 0.001 | **0.226**  *p* = 0.022 | **0.355**  *p* < 0.001 |
| IL-18 |  |  | 1.000 | **0.430**  *p* < 0.001 | **0.440**  *p* < 0.001 |
| IP-10 |  |  |  | 1.000 | **0.604**  *p* < 0.001 |
| MIG |  |  |  |  | 1.000 |

In bold those p-value <0.05.

**Supplemental Table 1**. Values of correlations between viral load and different cytokines.
